# The DDUP protein encoded by the DNA damage-induced CTBP1-DT lncRNA confers cisplatin resistance in ovarian cancer

**DOI:** 10.1038/s41419-023-06084-5

**Published:** 2023-08-26

**Authors:** Liangliang Ren, Xingrong Qing, Jihong Wei, Haixin Mo, Yuanji Liu, Yaofeng Zhi, Wenjie Lu, Mingzhu Zheng, Weijian Zhang, Yuan Chen, Yuejiao Zhang, Taijin Pan, Qian Zhong, Ronggang Li, Xin Zhang, Xiaohong Ruan, Ruyuan Yu, Jun Li

**Affiliations:** 1 Clinical Experimental Center, Jiangmen Key Laboratory of Clinical Biobanks and Translational Research, Jiangmen Central Hospital, Jiangmen, 529030 China; 2 Department of Gynecology, Jiangmen Central Hospital, Jiangmen, 529030 China; 3 Department of Biochemistry, Zhongshan school of medicine, Sun Yat-sen University, Guangzhou, 510080 China; 4 Department of Pathology, Jiangmen Central Hospital, Jiangmen, 529030 China; 5 Precision Medicine Institute, The First Affiliated Hospital, Sun Yat-sen University, Guangzhou, Guangdong, 510080 China

**Keywords:** Cancer therapeutic resistance, Biomarkers

## Abstract

Sustained activation of DNA damage response (DDR) signaling has been demonstrated to play vital role in chemotherapy failure in cancer. However, the mechanism underlying DDR sustaining in cancer cells remains unclear. In the current study, we found that the expression of the DDUP microprotein, encoded by the CTBP1-DT lncRNA, drastically increased in cisplatin-resistant ovarian cancer cells and was inversely correlated to cisplatin-based therapy response. Using a patient-derived human cancer cell model, we observed that DNA damage-induced DDUP foci sustained the RAD18/RAD51C and RAD18/PCNA complexes at the sites of DNA damage, consequently resulting in cisplatin resistance through dual RAD51C-mediated homologous recombination (HR) and proliferating cell nuclear antigen (PCNA)-mediated post-replication repair (PRR) mechanisms. Notably, treatment with an ATR inhibitor disrupted the DDUP/RAD18 interaction and abolished the effect of DDUP on prolonged DNA damage signaling, which resulted in the hypersensitivity of ovarian cancer cells to cisplatin-based therapy in vivo. Altogether, our study provides insights into DDUP-mediated aberrant DDR signaling in cisplatin resistance and describes a potential novel therapeutic approach for the management of platinum-resistant ovarian cancer.

## Introduction

Ovarian cancer is one of the deadliest gynecological malignancies worldwide, and 60–75% of women with ovarian cancer are diagnosed at an advanced stage at presentation [1, 2]. Despite significant efforts in the treatment of ovarian cancer over the past decades, the prognosis of patients with ovarian cancer remains very poor, with an overall 5-year survival rate of approximately 40% [3–5]. The poor prognosis has been primarily attributed to resistance to platinum-based chemotherapy, which is the standard chemotherapy regimen for ovarian cancer. Although approximately 80% of patients with ovarian cancer are sensitive to platinum-based chemotherapy drugs, nearly 25% patients with ovarian cancer relapse within less than 6 months of receiving platinum-based therapy, and approximately 75% patients suffer recurrence after 2 years. Therefore, understanding the mechanism underlying platinum-based chemotherapy resistance would aid in the development of novel therapeutic strategies for ovarian cancer.

Platinum-based chemotherapy drugs typically induce severe alterations in genomic DNA, including DNA single-strand breaks and double strand breaks (DSBs), which alter the structure of DNA, and can result in cellular injury and cell death due to apoptosis. Cells have developed diverse DNA damage response (DDR) mechanisms for promptly repairing the damaged DNA. Cells rapidly recruit several proteins to the chromatin sites surrounding the damaged DNA to initiate DDR, which coordinates the detection, maintenance, and repair of DNA damage signaling. The H2AX histone variant is rapidly phosphorylated in response to DNA damage by the phosphoinositide 3-kinase-related kinases (PIKK) family of kinases, including ataxia telangiectasia-mutated (ATM), ATM- and Rad3-related (ATR), and DNA-dependent protein kinase (DNA-PK), which bring large regions of phosphorylated H2AX (γ-H2AX) chromatin around the damaged DNA [6–8]. γ-H2AX is a marker of DNA damage and functions as a platform for the hierarchical recruitment and retention of various key DDR factors to form a complex which transmits the DNA damage signal for consequent DNA repair steps [9–11]. Therefore, sustaining the key DNA damage repair complexes at the DNA lesion sites would accelerate DNA damage repair.

Accumulating evidence over the past decades has elucidated the mechanisms by which DDR signaling promotes DNA repair in cells. It has been reported that homologous recombination (HR) repair and non-homologous end-joining repair play vital roles in repairing the primary DNA DSBs, which represent the most severe form of DNA damage [12, 13]. It has been demonstrated that the RAD51 recombinase and its paralogs, including RAD51C, RAD51B, RAD51D, and XRCC2, form two distinct protein complexes that contribute to HR repair in vivo. It has been recently reported that the E3 ubiquitin protein ligase, RAD18, which is a key DDR factor, exerts its DNA repair capacity via RAD51 paralogs to induce RAD51C-mediated HR repair and proliferating cell nuclear antigen (PCNA)-mediated post-replication repair (PRR) [11, 14, 15]. Consistent with the functions of RAD18 in DNA repair, previous studies have demonstrated that RAD18 plays vital roles in DNA damage-based chemo-radiotherapy resistance, including resistance to 5-fluorouracil (5-FU) and ionizing radiation [16, 17], and emphasize the essential role of RAD18 in DNA damage-based chemo-radiotherapy resistance. However, the regulatory mechanism underlying the retention of RAD18 at the sites of DNA damage remains to be elucidated.

The present study revealed that the expression of the CTBP1-DT lncRNA and the encoded protein, DDUP, was inversely correlated to the poor outcome of patients with ovarian cancer receiving cisplatin (CDDP) therapy. The study demonstrated that the CDDP-induced expression of DDUP, and not the expression of CTBP1-DT lncRNA, contributed to the repair of CDDP-induced DNA damage by retaining the RAD18/RAD51C and RAD18/PCNA complexes at the sites of DNA damage. Notably, treatment with an ATR inhibitor in combination with cisplatin (CDDP) drastically reversed DDUP-induced cisplatin resistance both in vitro and in vivo. Altogether, the findings provide novel insights into the role of DDUP in regulating cisplatin resistance and describes a promising therapeutic strategy for platinum-resistant ovarian cancer.

## Results

### Expression of CTBP1-DT lncRNA correlated with poor survival of cancer patients

**Fig. 1:**
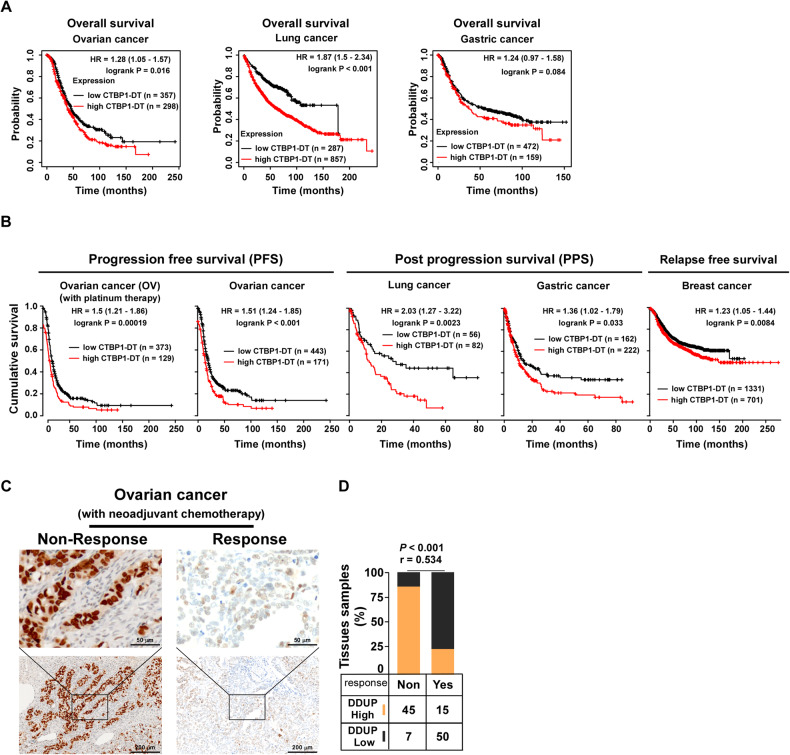
DDUP upregulation correlates with chemoresistance and poor prognosis of patients with ovarian cancer. **A**, **B** Online Kaplan-Meier plotter analysis revealed that patients with ovarian, lung, gastric, or breast cancer and high expression levels of CTBP1-DT had significantly shorter overall survival, progression-free survival, post progression survival, and relapse-free survival compared to those of patients with low CTBP1-DT expression (*P* < 0.05, log-rank test; *n* = biologically independent samples). **C** Representative images of DDUP expression in chemotherapy response and non-response ovarian cancer tissues (*n* = 117; left). **D** DDUP expression and neoadjuvant chemotherapy was strongly correlated in non-response and inversely correlated in response ovarian cancer tissues (*P* < 0.001, *r* = 0.534). The chi-square test was performed for statistical analysis.

Previous studies have reported that the elevation of DNA damage repair in cancer cells is associated with cancer chemoresistance and relapse [18–21]. In a recent study we demonstrated that the expression of the DDUP microprotein encoded by the CTBP1-DT lncRNA is induced by DNA damage, and plays a crucial role in DNA damage repair by sustaining the DDR signaling at DNA damage lesions [22]. However, the clinical significance of the CTBP1-DT lncRNA in human cancers remains to be clearly elucidated to date. Analysis of the publicly available online KaplanMeier plotter dataset [23] revealed that patients with ovarian, lung, or gastric cancer and high expression of CTBP1-DT lncRNA had significantly shorter overall and progression-free survival compared to those of patients with low expression of CTBP1-DT lncRNA (Fig. 1A, B). Notably, the expression of CTBP1-DT lncRNA was inversely correlated with shorter progression-free and overall survival in a cohort of ovarian cancer patients who received platinum-based chemotherapy (Fig. 1B). This suggested that the upregulation of CTBP1-DT lncRNA could be correlated with the failure of chemotherapy and cancer relapse.

The development of platinum-based chemotherapy resistance is the primary reason underlying tumor relapse [24]. It has been reported that approximately 25% of patients with ovarian cancer exhibit recurrence within 6 months of treatment with standard platinum therapy, and more than 75% patients with ovarian cancer exhibit platinum-based chemotherapy resistance and suffer recurrence, resulting in a 5-year survival rate of approximately only 30% [25–27]. Therefore, ovarian cancer was selected as the disease model in this study. We further examined the correlation between platinum-based chemotherapy response and the expression of DDUP protein, encoded by the CTBP1-DT lncRNA, using immunohistochemistry (IHC) assays. The response to platinum-based chemotherapy was classified according to RECIST1.1 into the response category, which included complete response and partial response, and the non-response category, which included progressive disease and stable disease [28]. As depicted in Fig. 1C, the expression of DDUP in the non-response group was significantly higher than that in the response group (*P* < 0.001, *r* = 0.534). Altogether, these results suggested that the expression of DDUP is associated with the poor outcome of patients with ovarian cancer and could serve as an independent predictor of cisplatin response.

### DNA damage induced the expression of DDUP but not CTBP1-DT lncRNA

**Fig. 2:**
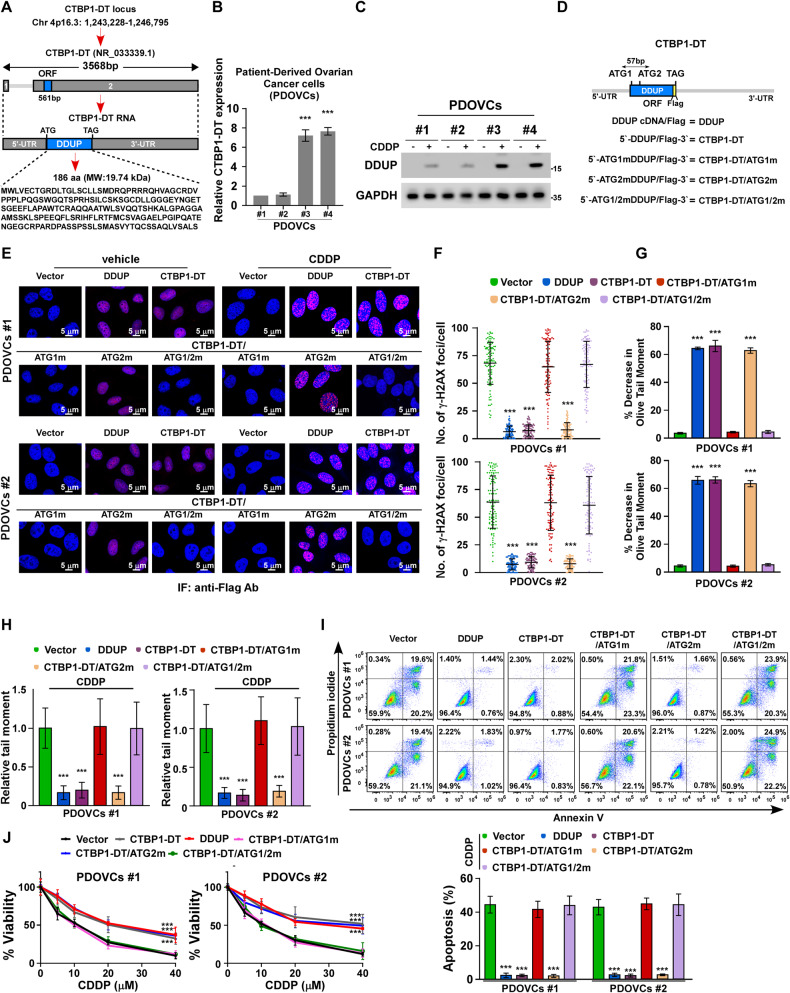
DDUP overexpression promoted CDDP resistance in PDOVCs. **A** LncRNA CTBP1-DT is located on chromosome Chr 4p16.3 (1,243,228−1,246,795). The open reading frame (ORF) for the DDUP protein located in exon 2 of CTBP1-DT, and the molecular weight (MW) of DDUP protein is 19.74 kDa. **B** Real-time PCR analysis of CTBP1-DT expression in chemosensitive PDOVCs#1 and PDOVCs#2 and chemoresistant PDOVCs#3 and PDOVCs#4. *GAPDH* served as the control. **C** The expression of DDUP in PDOVCs treated with the vehicle or CDDP (12.3 μM) was detected by IB analysis. GAPDH served as the loading control. **D** Diagram of the indicated lncRNA CTBP1-DT constructs, including wild-type (DDUP), ATG1 mutation (CTBP1-DT/ATG1m), ATG2 mutation (CTBP1-DT/ATG2m), and double ATG site mutation (CTBP1-DT/ATG1/2m; ATG mutated to ATT). **E** IF staining for analyzing the expression of DDUP using an anti-Flag antibody against PDOVCs#1 and PDOVCs#2 transfected with CTBP1-DT lncRNA constructs following treatment with the vehicle or CDDP (12.3 μM, 1 h). Scale bar = 5 μm. **F** Quantification of γ-H2AX foci in PDOVCs transfected with CTBP1-DT lncRNA constructs following treatment with CDDP (12.3 μM, 1 h). At least 100 cells were counted. **G** PDOVCs#1 and PDOVCs#2 cells were treated with CDDP (12.3 μM) for 1 h and analyzed for cross-linking 7 h post-treatment. The average olive tail moment of 100 comets was calculated. The percentage decrease in olive tail moment in comparison with untreated samples was then calculated (please see Materials and Methods section). **H** Quantification of damaged DNA in the cells transfected with CTBP1-DT lncRNA constructs following treatment with CDDP (12.3 μM, 4 h after treatment), as determined by neutral comet assay (*n* = 100). **I** FACS analysis of the apoptotic rate of indicated cells treated with CDDP (12.3 μM, 24 h) using Annexin V-FITC/PI apoptosis detection kits. **J** The survival rate of the indicated cells was first treated with various concentrations of CDDP for 24 h and further cultured for 96 h in the fresh medium, and then determined by MTT assays. The error bars represent the mean ± standard deviation (SD) of data from three independent experiments; *P < 0.05, **P < 0.01, ***P < 0.001.

The human gene CTBP1-DT is located on chromosome Chr 4p16.3 (1,243,228–1,246,795), which it’s transcript lncRNA CTBP1-DT (NR_033339.1) contains two exons. The open reading frame (ORF) for DDUP protein is 561 bp in the 2nd exon of the lncRNA CTBP1-DT, which encodes a 186-amino-acid protein (Molecular weight: 19.74 kDa) (Fig. 2A). In order to investigate whether the CTBP1-DT lncRNA and/or DDUP contributes to cisplatin resistance, patient-derived ovarian cancer cells (PDOVCs), which are expected to resemble ovarian cancer cells in clinical tumor tissues, were isolated from two CDDP-sensitive ovarian cancer tissues and denoted as PDOVCs#1 and PDOVCs#2, and from two CDDP-resistant ovarian cancer tissues and denoted as PDOVCs#3 and PDOVCs#4. As depicted in Fig. 2B, the expression of CTBP1-DT lncRNA was significantly higher in CDDP-resistant PDOVCs#3 and PDOVCs#4 than in CDDP-sensitive PDOVCs#1 and PDOVCs#2. Interestingly, the results of immunoblotting (IB) analysis demonstrated that DDUP was not expressed in all the four PDOVCs in the absence of CDDP treatment (Fig. 2C). However, treatment with CDDP dramatically induced the expression of DDUP protein in all four PDOVCs, and the levels of DDUP were higher in the CDDP-resistant PDOVCs#3 and PDOVCs#4 than in the CDDP-sensitive PDOVCs#1 and PDOVCs#2 (Fig. 2C). These findings were consistent with a previous study which reported that DDUP is induced in response to treatment of DNA damage [22].

In order to further determine the effect of DDUP protein or the host gene encoding the CTBP1-DT lncRNA on CDDP resistance, we constructed a series of plasmids encoding the CTBP1-DT lncRNA, which either expressed or did not express the DDUP protein (Fig. 2D). The plasmids included DDUP cDNA (DDUP) and CTBP1-DT, and three mutated ATG plasmids, namely, CTBP1-DT/ATG1m, CTBP1-DT/ATG2m, and CTBP1-DT/ATG1/2m, which comprised two closely located ATG codons in the open reading frame (ORF). Although the results of real-time polymerase chain reaction (PCR) indicated that the RNA expression of the plasmids in PDOVCs#1 and PDOVCs#2 treated with the vehicle or CDDP was nearly similar (Supplementary Fig. 1A), immunofluorescence (IF) staining with an anti-Flag antibody revealed that Flag-tagged DDUP was expressed in the nucleus of PDOVCs transfected with DDUP, CTBP1-DT, and CTBP1-DT/ATG2m in the absence of CDDP treatment, but not in PDOVCs#1 and PDOVCs#2 transfected with CTBP1-DT/ATG1m and CTBP1-DT/ATG1/2m (Fig. 2E). The PDOVCs#1 and PDOVCs#2 transfected with DDUP, CTBP1-DT, and CTBP1-DT/ATG2m had clear DDUP foci in response to CDDP treatment (Fig. 2E). These results further confirmed that the expression of DDUP could be induced in cells with DNA damage.

### Upregulation of DDUP, and not CTBP1-DT lncRNA, conferred CDDP resistance

The effect of CTBP1-DT lncRNA and/or DDUP protein on CDDP-induced DNA damage repair was subsequently examined. Cisplatin, one of the most widely used and effective anticancer agents, targets the DNA by inducing DNA adducts and crosslinks, then leading to endogenous DNA damage (single- and double-strand breaks), which consequently resulted in activation of the DDR and apoptotic cell death [18, 29, 30]. Consistent with the upregulation of DDUP expression induced by CDDP, we observed that overexpression of either DDUP, or CTBP1-DT, or CTBP1-DT/ATG2m not only reduced the CDDP-induced DNA crosslinks but also decreased the CDDP-induced DNA damage analyzed via alkaline denaturing and neutral comet assays and γ-H2AX foci staining assay (Fig. 2F–H). However, the levels of CDDP-induced DNA damage were nearly same in PDOVCs#1 and PDOVCs#2 transfected with CTBP1-DT/ATG1m and CTBP1-DT/ATG1/2m (Fig. 2F–H). These findings indicated that the DDUP protein encoded by the CTBP1-DT lncRNA, and not the CTBP1-DT lncRNA, plays an important role in DNA damage repair.

We subsequently determined the relevance of DDUP upregulation in CDDP resistance. The five abovementioned CTBP1-DT constructs transduced-PDOVCs#1 and -PDOVCs#2 were treated with CDDP, an IC50 value of 12.3 μM (Supplementary Fig. 2B, C), and further subjected to FACS analysis using an Annexin V-FITC/PI apoptosis detection kit. As depicted in Fig. 2I, the increase in the apoptotic rate of PDOVCs#1 and PDOVCs#2 transfected with DDUP, CTBP1-DT, or CTBP1-DT/ATG2m following treatment with CDDP was significantly lower than that of the control cells; however, the apoptotic rate of PDOVCs transfected with CTBP1-DT/ATG1m or CTBP1-DT/ATG1/2m was similar to that of the control cells. Notably, we observed that the CTBP1-DT constructs that ectopically expressed DDUP significantly increased cellular survival following treatment with CDDP, while the constructs that did not encode DDUP did not increase cell survival (Fig. 2J). Altogether, the results demonstrated that the upregulation of DDUP, and not CTBP1-DT lncRNA, enhanced the capability of ovarian cancer cells to repair damaged DNA, which resulted in resistance to CDDP.

### CRISPR/Cas9-mediated knockout (KO) of DDUP increases CDDP sensitivity

**Fig. 3:**
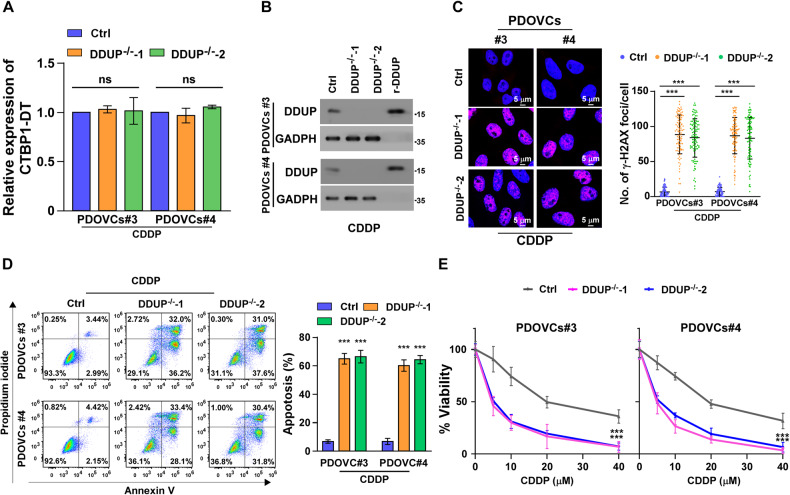
DDUP KO increased the sensitivity of PDOVCs to CDDP treatment. **A** The expression of CTBP1-DT in the CDDP (12.3 μM) -treated control cells and DDUP^-/-^ PDOVCs#3 and PDOVCs#4 was determined by real-time PCR. *GAPDH* served as the control. **B** IB analysis of endogenous DDUP expression in the CDDP (12.3 μM) -treated control and DDUP^-/-^ PDOVCs#3 and PDOVCs#4. GAPDH served as the loading control. **C** Representative images (left) images and quantification (right) of γ-H2AX foci in the CDDP-treated (12.3 μM, 1 h) control and DDUP^-/-^ PDOVCs#3 and PDOVCs#4. At least 100 cells were counted. **D** The rate of apoptosis of the CDDP-treated (12.3 μM, 24 h) control and DDUP^-/-^ PDOVCs#3 and PDOVCs#4 was determined by FACS analysis. **E** The survival rates of the control and DDUP^-/-^ cells treated with various concentrations of CDDP for 24 h and further cultured for 96 h in the fresh medium, which were determined by MTT assays. The error bars represent the mean ± SD of data from three independent experiments; *P < 0.05, **P < 0.01, ***P < 0.001.

In order to confirm that the DDUP protein, and not the CTBP1-DT lncRNA, confers CDDP resistance in ovarian cancer, a CRISPR/Cas9-KO system was used to establish DDUP KO (DDUP^-/-^)-PDOVCs#3 and -PDOVCs#4. As depicted in Fig. 3A, the results of real-time PCR assay demonstrated that the expression of CTBP1-DT lncRNA in DDUP^-/-^ PDOVCs#3 and PDOVCs#4 was comparable to that of the control cells following CDDP treatment; However, treatment with CDDP did not increase the expression of DDUP protein in DDUP^-/-^ PDOVCs#3 and PDOVCs#4, as revealed by IB studies (Fig. 3B). These results indicated the successful establishment of the DDUP^-/-^ PDOVCs#3 and PDOVCs#4.

We subsequently examined the effect of DDUP KO on CDDP treatment using a series of functional experiments. Although DDUP KO had no effect on cell survival (Supplementary Fig. 2A), the DNA damage induced by CDDP treatment in DDUP^-/-^ PDOVCs#3 and PDOVCs#4 was much higher than that of the control cells, as indicated by γ-H2A foci staining (Fig. 3C). These results further confirmed that the DDUP protein, and not the CTBP1-DT lncRNA, conferred resistance to CDDP in ovarian cancer. As cell death is primarily attributed to apoptosis induced by DNA damage, we investigated the effect of DDUP KO on CDDP-induced apoptosis. As depicted in Fig. 3D, the apoptotic rates of DDUP^-/-^ PDOVCs#3 and PDOVCs#4 were significantly higher than that of the control cells following treatment with CDDP. The results of cell viability analysis further demonstrated that DDUP KO inhibited the resistance of DDUP^-/-^ PDOVCs#3 and PDOVCs#4 to CDDP, as indicated by the significant reduction in the number of cells (Fig. 3E). Altogether, these results indicated that DDUP KO renders sensitivity to CDDP treatment.

### DDUP mediated DNA damage repair by sustaining DDR signaling

**Fig. 4:**
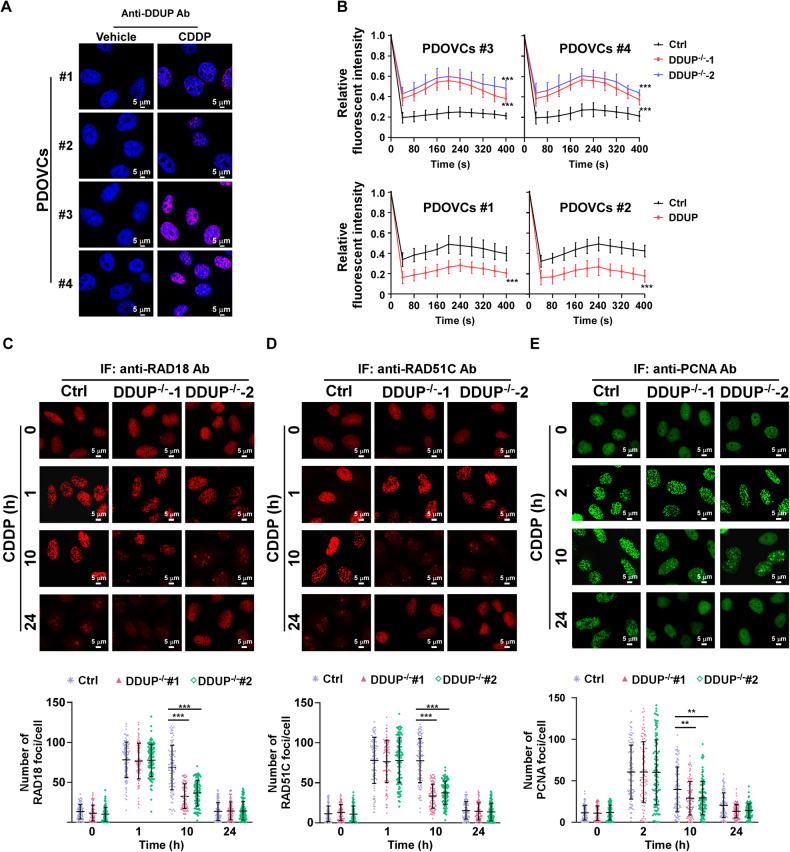
DDUP KO reduced the effect of RAD18 on DNA damage repair. **A** The expression of DDUP in PDOVCs treated with the vehicle or CDDP (12.3 μM, 1 h) was determined by IF staining. **B** Quantitative FRAP analysis of GFP-RAD18 in the GFP-RAD18-transfected control and DDUP^-/-^ PDOVCs treated with CDDP followed by subsequent recovery for the durations indicated. Representative images (left) and time course (right) of the formation of CDDP (12.3 μM) -induced RAD18 foci (**C**), RAD51C (**D**), and PCNA (**E**) foci in the control and DDUP^-/-^ PDOVCs following recovery for the indicated durations. At least 100 cells were counted. The error bars represent the mean ± SD of three independent experiments; *P < 0.05, **P < 0.01, ***P < 0.001.

Consistent with the results of IB analysis (Fig. 2B), the results of IF staining revealed that the endogenous DDUP protein could not be detected in the PDOVCs that were not treated with CDDP; however, treatment with CDDP drastically induced DDUP expression and the formation of DDUP foci (Fig. 4A). In our previous study we demonstrated that DDUP-induced DNA damage repair occurs via RAD18/RAD51C-mediated HR and RAD18/PCNA-mediated PRR mechanisms. We subsequently examined the dynamics of CDDP-induced RAD18 foci in PDOVCs by fluorescence recovery after photo bleaching (FRAP) assays. As depicted in Fig. 4B, DDUP KO in PDOVCs#3 and PDOVCs#4 significantly increased the recovery rate of the signal from GFP-RAD18 foci (approximately 56%) compared to that of the control cells, which had a recovery rate of approximately 23%. However, DDUP overexpression significantly reduced the recovery rate of the signal from GFP-RAD18 foci in PDOVCs#1 and PDOVCs#2. The findings revealed that DDUP plays a crucial role in retaining RAD18 at the sites of DNA damage (Fig. 4B).

The effect of DDUP in retaining RAD18, RAD51C, and PCNA at the sites of DNA damage was subsequently examined. To this end, CDDP-treated control cells and DDUP-KO cells were subjected to IF assays at different time points using anti-RAD18, anti-RAD51C, or anti-PCNA antibodies. As depicted in Fig. 4C, D, the peaks of the RAD18 and RAD51C foci induced by DNA damage appeared simultaneously in the control and DDUP-KO cells, at approximately 1 h after treatment with CDDP. These results indicated that DDUP was not involved in the formation of RAD18 and RAD51C foci induced by DNA damage. However, the RAD18 and RAD51C foci rapidly disappeared in DDUP-KO cells and reached background levels in less than 10 h, while the peaks of the RAD18 and RAD51C foci disappeared slowly in the control cells and reached background levels after approximately 24 h (Fig. 4C, D). Similar patterns were observed for the PCNA foci that rapidly disappeared in DDUP-KO cells treated with CDDP (Fig. 4E). Altogether, the findings indicated that DDUP-induced DNA damage repair occurs via the retention of DDR factors at the sites of DNA damage.

### Inhibition of ATR activity reversed DDUP-induced CDDP-resistance in vitro

**Fig. 5:**
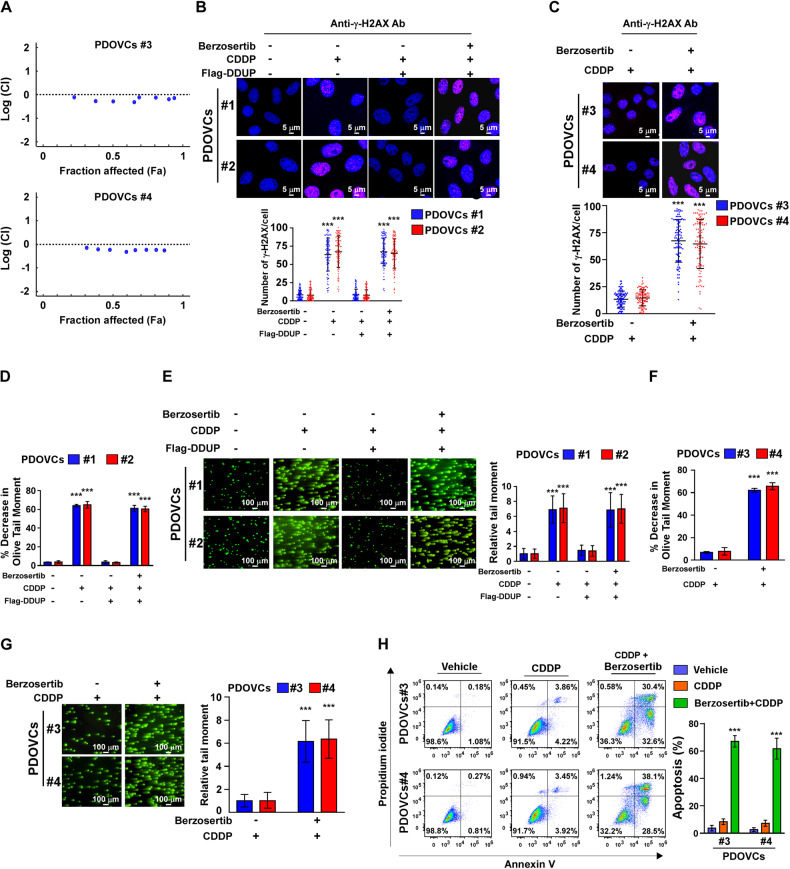
ATR is essential for DDUP-induced chemoresistance in ovarian cancer cells. **A** Logarithmic combination index (CI) plot for the combination of CDDP (12.3 μM) and different concentrations of Berzosertib (10–1280 nM) in the indicated cells. The combination of CDDP (12.3 μM) with Bezosertib (80 nM) was calculated to display most anti-proliferation effect on DDUP high-expressed PDOVC#3 and PDOVC#4 using Chou-Talalay method. **B** Representative images (upper) and quantification (bottom) of γ-H2AX foci in vector- or DDUP-transduced PDOVCs#1 and PDOVCs#2 treated with either CDDP (12.3 μM) alone or CDDP (12.3 μM) plus Berzosertib (80 nM) for 1 h. At least 100 cells were counted. Scale bar = 5 μm. **C** Representative images (upper) and quantification (bottom) of γ-H2AX foci in PDOVCs#3 and PDOVCs#4 following treatment with CDDP (12.3 μM) alone or CDDP (12.3 μM) plus Berzosertib (80 nM) for 1 h. At least 100 cells were counted. Scale bar = 5 μm. **D** PDOVCs#1 and PDOVCs#2 cells were treated with CDDP (12.3 μM) or combination of CDDP (12.3 μM) and Berzosertib (80 nM) for 1 h and analysed for cross-linking 7 h post-treatment, as determined by alkaline denaturing comet assay. The average olive tail moment of 100 comets was calculated. The percentage decrease in olive tail moment in comparison with untreated samples was then calculated (please see Materials and Methods section). **E** Representative images (left) and quantification (right) of the tail moments of PDOVCs#1 and PDOVCs#2 using comet assays following treatment with CDDP (12.3 μM) alone or CDDP (12.3 μM) plus Berzosertib (80 nM) for 4 h, as determined by neutral comet assay. At least 100 cells were counted. Scale bar = 100 μm. **F** PDOVCs#3 and PDOVCs#4 cells were treated with CDDP (12.3 μM) or combination of CDDP (12.3 μM) and Berzosertib (80 nM) for 1 h and analyzed for cross-linking 7 h post-treatment, as determined by alkaline denaturing comet assay. The average olive tail moment of 100 comets was calculated. The percentage decrease in olive tail moment in comparison with untreated samples was then calculated (please see Materials and Methods section). **G** Representative images (left) and quantification (right) of the tail moments of PDOVCs#3 and PDOVCs#4 following treatment with CDDP (12.3 μM) alone or CDDP (12.3 μM) plus Berzosertib (80 nM) for 4 h, as determined by neutral comet assay. At least 100 cells were counted. Scale bar = 100 μm. **H** The rate of apoptosis of the indicated PDOVCs (#3 to #4) was determined by FACS analysis following treatment with CDDP (12.3 μM) alone or CDDP (12.3 μM) plus Berzosertib (80 nM) for 24 h. The error bars represent the mean ± SD of data from three independent experiments; *P < 0.05, **P < 0.01, ***P < 0.001.

In our previous study we demonstrated that the ATR-mediated phosphorylation of DDUP is necessary for DDUP-mediated DNA damage repair in HeLa cells [22]. We then examined whether inhibition of ATR activity using Berzosertib, an ATR inhibitor, could reverse DDUP-induced CDDP resistance. To this end, Chou-Talalay method using CalcuSyn software [31, 32], which has been widely accepted to study synergistic drug interactions [33–36], was used to determine the best anti-tumor effect using CDDP (12.3 μM) and different concentrations of ATR inhibitor Bezosertib in DDUP high-expressed PDOVC#3 and PDOVC#4. The combination index (CI) theorem of Chou-Talalay offers quantitative definition for additive effect (CI = 1), synergism (CI < 1), and antagonism (CI > 1) in drug combination. As shown in Fig. 5A, the combination of CDDP (12.3 μM) with Bezosertib (80 nM) displayed most anti-proliferation effect on DDUP high-expressed PDOVC#3 (CI = 0.471) and PDOVC#4 (CI = 0.475). Therefore, the combination of CDDP (12.3 μM) with Bezosertib (80 nM) was used for treatment in the followed studies.

Furthermore, we found that DDUP overexpression in PDOVCs#1 and PDOVCs#2, which have low DDUP expression levels, drastically reduced the induction of γ-H2AX following CDDP treatment (Fig. 5B). These findings further confirmed the role of DDUP in DNA damage repair. However, combined treatment with Berzosertib and CDDP significantly abrogated the inhibitory effect of DDUP upregulation on the induction of γ-H2AX by CDDP treatment in the DDUP-overexpressing cells or DDUP high-expressed cells (Fig. 5B, C). Furthermore, the alkaline denaturing and neutral comet assays showed that co-treatment with CDDP and Berzosertib increased the CDDP-induced DNA crosslinks and also DNA damage in the DDUP-overexpressing cells, compared to those of cells treated with CDDP alone (Fig. 5D, E). These findings suggested that the inhibition of ATR activity impaired DDUP-mediated DNA damage repair. Consistent with this hypothesis, we observed that co-treatment with CDDP and Berzosertib resulted in increasing level of either DNA crosslinks or DNA damage in DDUP high-expressed PDOVCs#3 and PDOVCs#4 compared to that of cells treated with CDDP alone (Fig. 5F, G).

The effect of the CDDP/Berzosertib combination therapy on DDUP-induced resistance was subsequently assessed in vitro. The findings revealed that compared to CDDP monotherapy, the CDDP/Berzosertib combination therapy significantly increased the apoptotic rate of PDOVCs#3 and PDOVCs#4, which express high levels of DDUP (Fig. 5H). Altogether, these results demonstrated that the inhibition of ATR activity reversed the CDDP resistance induced by DDUP in vitro.

### CDDP/Berzosertib combination therapy impaired carboplatin resistance in ovarian cancer cells with high DDUP expression in vivo

**Fig. 6:**
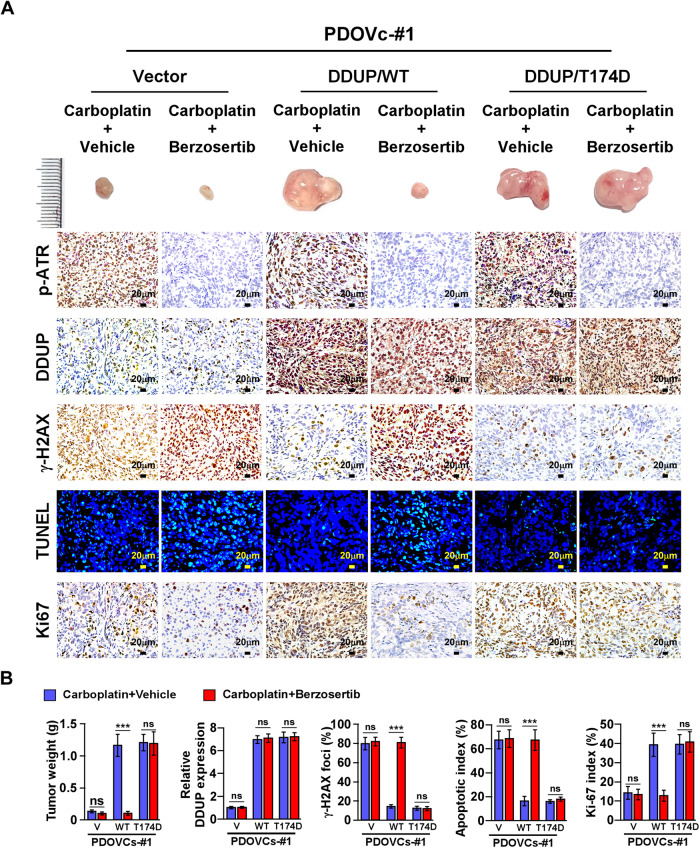
Upregulation of DDUP confers carboplatin resistance to ovarian cancer in vivo. **A**, **B** PDOVCs#1 stably expressing DDUP/WT or DDUP/mutant were subcutaneously inoculated into immunodeficient mice, following treatment with carboplatin (50 mg/kg) alone or carboplatin ((50 mg/kg) plus Berzosertib (60 mg/kg). Six mice were randomly assigned to each group. **A** Representative images of xenograft tumors and results of IHC and TUNEL staining. **B** Quantification of the weights of the xenograft tumors, DDUP, γ-H2AX, and Ki-67 staining, and the apoptotic index of the xenograft tumors treated with the indicated chemotherapy agents. The error bars represent the mean ± SD of data from three independent experiments; *P < 0.05, **P < 0.01, ***P < 0.001.

It has been reported that the ATR-mediated phosphorylation of DDUP is necessary for DDUP-induced DNA damage repair via the formation of the γ-H2AX/DDUP/RAD18 complex [22]. We therefore examined the effect of DDUP dysregulation on carboplatin resistance in an in vivo animal model in the presence or absence of the ATR inhibitor, Berzosertib. We established the DDUP/WT and DDUP/T174D plasmids, which mimic DDUP phosphorylation, and PDOVCs#1 was subsequently transfected with these plasmids. The PDOVCs were subcutaneously inoculated into highly immunodeficient NOD-SCID IL-2rγ^−/−^ (NSG) mice. As depicted in Fig. 6A, B, the tumors overexpressing DDUP/WT exhibited higher resistance to carboplatin treatment, as indicated by the increased tumor volume, higher Ki-67 index, and decreased γ-H2AX and TUNEL signals. However, Berzosertib nearly abolished the strong carboplatin resistance induced by DDUP overexpression. Interestingly, the tumors formed by PDOVCs#1 transfected with DDUP/T174D exhibited higher resistance to carboplatin monotherapy and the carboplatin/Berzosertib combination therapy (Fig. 6A, B). These findings further supported the notion that the ATR-mediated phosphorylation of DDUP is necessary for DNA damage repair induced by DDUP.

**Fig. 7:**
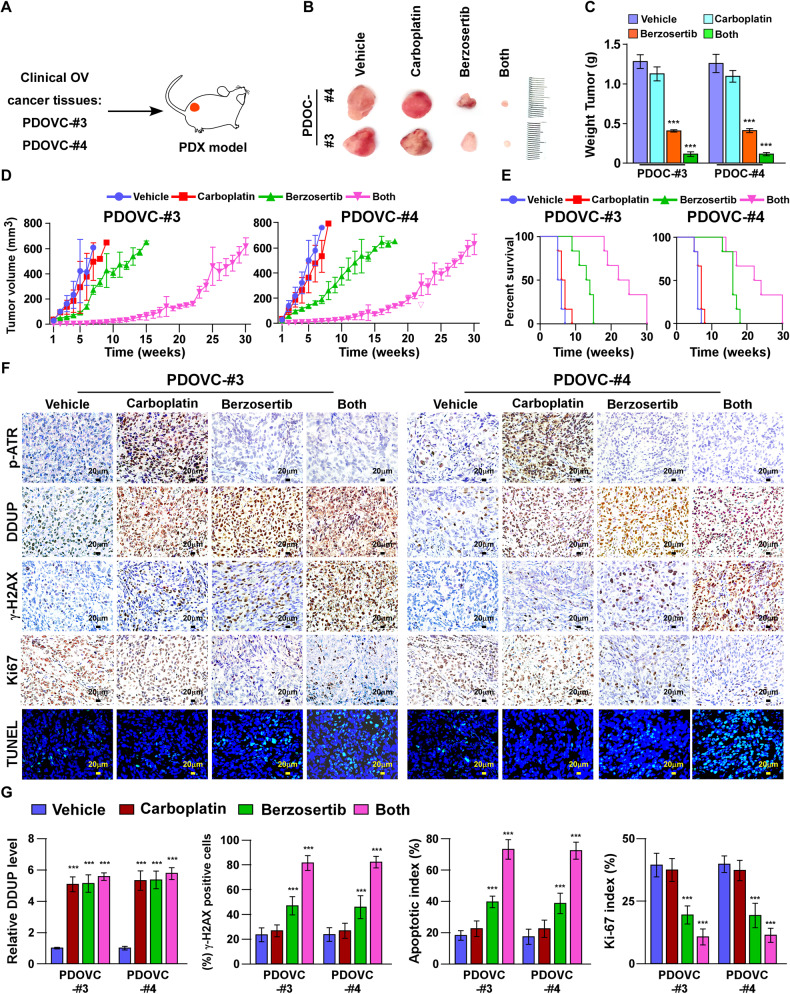
Berzosertib enhances the sensitivity of ovarian cancer cells with high DDUP expression to carboplatin in PDX-model. **A** A PDX model was established by inoculating with two freshly collected primary ovarian cancer tissues obtained clinically (PDOVC#3 and PDOVC#4). Six mice were randomly assigned to each group. **B** Representative images of the xenograft tumors in response to the indicated treatments with the vehicle, carboplatin (50 mg/kg), Berzosertib (60 mg/kg), or carboplatin (50 mg/kg) plus Berzosertib (60 mg/kg); *n* = 6/group. **C** Weights of the xenograft tumors treated with different chemotherapeutic agents. **D** Tumor volumes measured on the indicated days. **E** Kaplan−Meier survival curves for the indicated mice (*n* = 6). **F** Representative images of IHC staining of DDUP, γ-H2AX, and Ki-67, and representative images of TUNEL assays. **G** Quantification of DDUP, γ-H2AX, and Ki-67 staining and the apoptotic index for the xenograft tumors receiving the indicated treatments. The error bars represent the mean ± SD of data from three independent experiments; *P < 0.05, **P < 0.01, ***P < 0.001.

**Fig. 8:**
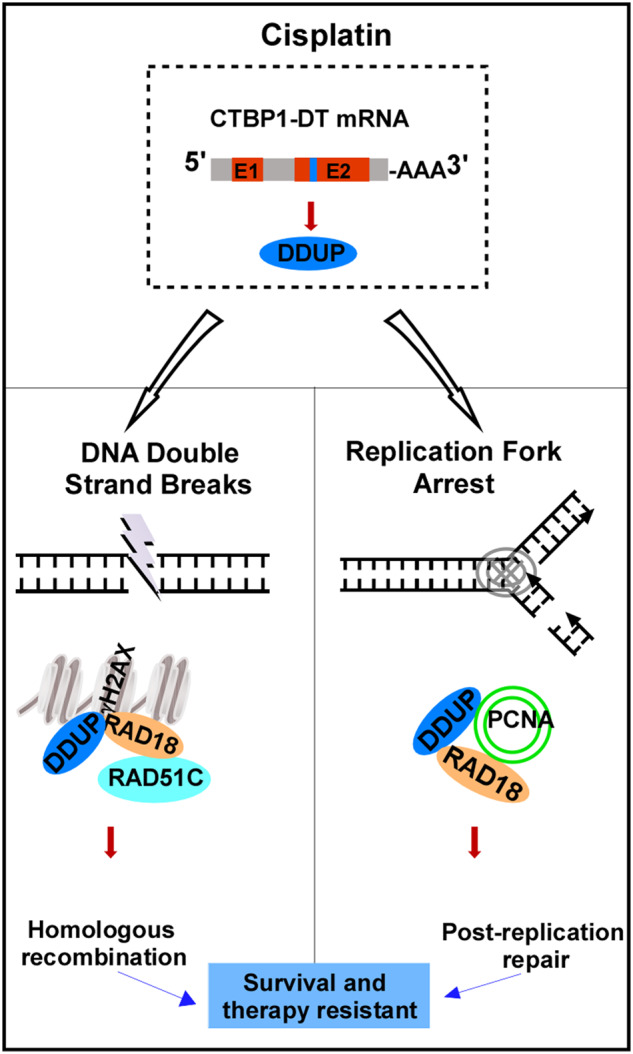
Hypothetical model. Schematic diagram illustrating that DDUP encoded by CTBP1-DT lncRNA confers cisplatin resistance in ovarian cancer through dual RAD51C-mediated homologous recombination (HR) and proliferating cell nuclear antigen (PCNA)-mediated post-replication repair (PRR) mechanisms.

We subsequently examined the therapeutic effect of the carboplatin/Berzosertib combination therapy on ovarian cancer using an in vivo patient-derived xenograft (PDX) model established using two clinical ovarian cancer tissues (PDOVC-#3 and PDOVC-#4; Fig. 7A), which exhibited higher DDUP expression following DNA damage. As depicted in Fig. 7B–E, both PDOVC-#3/PDX and PDOVC-#4/PDX displayed obvious resistance to carboplatin therapy, and the tumor volumes and median overall survival were similar to those of the control. However, the carboplatin/Berzosertib combination therapy markedly reduced the volume of tumors in PDOVC-#3/PDX and PDOVC-#4/PDX mice, which had higher γ-H2AX and apoptotic indices but lower Ki-67 indices. The median overall survival of PDOVC-#3/PDX and PDOVC-#4/PDX mice was significantly higher than that of mice receiving carboplatin or Berzosertib monotherapy (Fig. 7F, G). Altogether, the results demonstrated that the combination of a platinum-based chemotherapy agent with an ATR inhibitor improves therapeutic outcomes in an ovarian cancer model, and could represent a potential novel strategy for overcoming the clinical recurrence of ovarian cancer (Fig. 8).

## Discussion

The poor survival rate of ovarian cancer is primarily attributed to platinum-based chemotherapy resistance [37]. Clinically, 60–80% of patients with ovarian cancer have a preliminary response to platinum-based chemotherapy but most patients eventually suffer from recurrence due to platinum-based chemotherapy resistance [38]. Therefore, understanding the mechanism underlying platinum-based resistance in ovarian cancer is crucial for developing potential and effective therapeutic regimens. We have previously reported that the CTBP1-DT lncRNA, which encodes the DDUP microprotein, is rapidly upregulated following DNA damage, and aids in sustaining the DDR signal at the DNA lesions [22]. However, the clinical significance of DDUP in chemotherapy response in the clinics remains unclear. In this study, we observed that the expression of DDUP was strongly associated with CDDP resistance in ovarian cancer and was inversely correlated with the survival rate of patients with ovarian cancer receiving platinum-based chemotherapy. Notably, treatment with an ATR inhibitor abolished DDUP-induced DNA damage repair and protection against apoptosis, leading to the development of CDDP-resistant ovarian cancer cells that were more vulnerable to platinum-based chemotherapeutic agents [39, 40]. Altogether, the findings highlight the importance of DDUP as a predictive biomarker of CDDP resistance and possibly represent a potential strategy for the treatment of CDDP-resistant ovarian cancer.

The ability of cancer cells to sense and repair damaged DNA impairs the efficacy of chemotherapeutic agents, suggesting that the components of the DDR pathway, including sensors, transducers, and effectors, might contribute to the acquired or inherent resistance of cancer cells to DNA damage agents used clinically. Cross-talks between components of the DDR signaling pathway and resistance to chemoradiation therapy is an attractive field of research in cancer therapy; however, the mechanisms underlying the dysregulation of DDR in cancer, which promotes chemoresistance, remain largely unknown. In this study, we observed that the CDDP-induced upregulation of DDUP promoted the ability of ovarian cancer cells to repair the DNA damage induced by treatment with CDDP. The study further demonstrated that DDUP could interact with and retain RAD18 at the sites of DNA damage. This resulted in a sustained DDR signal mediated via dual RAD51C-mediated HR and PCNA-mediated PRR, which are mediated by interactions with RAD18. These findings elucidated a plausible mechanism underlying the CDDP resistance mediated by a sustained DDR.

Recent accumulating evidence confirmed that non-coding RNAs and their encoded proteins can serve as novel potential therapeutic targets for various types of carcinomas [41–43]. For instance, Li et al. demonstrated that the MIAC micropeptide encoded by an lncRNA inhibits the progression and metastasis of renal carcinoma by deactivating the EGEG/EGFR signaling pathway [44]. The 184-residue cGGNBP2 microprotein encoded by a circRNA plays a positive regulatory role in modulating the IL-6/STAT3 signaling pathway by directly interacting with and phosphorylating STAT3, and could serve as an auxiliary target for the development of clinical treatments for intrahepatic cholangiocarcinoma by targeting the IL-6/STAT3 pathway [45]. Furthermore, the study by Peng and coworkers demonstrated that the 295-residue AXIN1 protein encoded by circAXIN1 promotes the progression of gastric cancer by activating the Wnt signaling pathway [46]. These findings suggested that targeting non-coding RNAs and their encoded proteins could provide a novel strategy for the diagnosis and treatment of human cancers. It has been previously reported that the CTBP1-DT lncRNA is upregulated in a variety of human cancer types, including high-grade serous ovarian cancer, hepatocellular carcinoma, gliomas, and breast cancer [47–50]. We have recently demonstrated that treatment with CDDP can induce the translation of the CTBP1-DT lncRNA into DDUP, which confers resistance to ovarian cancer cells by enhancing the DNA damage repair ability. In this study, we observed that the expression of DDUP was markedly upregulated in ovarian cancer cells with CDDP resistance, and patients with higher DDUP expression had shorter overall and disease-free survival time following platinum-based chemotherapy, while patients with lower DDUP expression had better prognosis. In our previous study, we demonstrated that the ATR-mediated phosphorylation of DDUP is essential for DDUP-induced DNA damage repair. In this study, we further demonstrated that combination therapy with the ATR inhibitor, Berzosertib, which is an intravenously administered small molecule with promising anti-tumor activity in multiple phase I/II clinical trials, significantly promoted the sensitivity of ovarian cancer cells to CDDP resistance. Therefore, the study provides further evidence regarding lncRNA-mediated CDDP resistance, and describes a novel mechanism of inducible CDDP resistance in ovarian cancer.

## Materials and methods

### Patient derived cells and cell culture

Patient derived ovarian cancer cell #1, patient derived ovarian cancer cell #2, patient derived ovarian cancer cell #3, and patient derived ovarian cancer cell #4 were isolated from four clinical ovarian cancer tissues which were histopathologically and clinically diagnosed at Jiangmen Central Hospital (Jiangmen, Guangdong, China). Each patient signed consent and was able to withdraw her consent at any time and this study was approved by the Institutional Research Ethics Committee of the Jiangmen Central Hospital. All patient derived cells were prepared from fresh ovarian cancer samples as previously described [51] and subjected to mycoplasma contamination.

### Tissue specimens and Immunohistochemistry (IHC) analysis

The 117 paraffin-embedded surgical ovarian cancer samples with neoadjuvant chemotherapy were histopathologically and clinically diagnosed at Jiangmen Central Hospital (Jiangmen, Guangdong, China) or the First Affiliated hospital of Sun Yat-sen University (Guangzhou, Guangdong, China). This basic research study complied with all relevant ethical regulations involving human participants. Prior patient consent and approval were obtained from the Institutional Research Ethics Committee of the Jiangmen Central Hospital and the First Affiliated hospital of Sun Yat-sen University (Approval number: 2022-121).

IHC analysis was carried out to determine altered protein expression in indicated paraffin-embedded ovarian cancer tissues and followed by anti-DDUP antibody (1:100), overnight at 4 °C. According to the histopathological features and patient data of the tissues, the degree of immunostaining of formalin-fixed, paraffin-embedded sections was reviewed and scored separately by two independent pathologists. The scores were determined by the proportion of positively stained tumor cells coupled with the intensity of staining. The scores given by the two independent pathologists were combined into a mean score for further comparative evaluation. Tumor cell proportions were scored as follows: 0, no positive tumor cells; 1, <10% positive cells; 2, 10–35% positive tumor cells; 3, 35–75% positive tumor cells; 4, >75% positive tumor cells. Staining intensity was graded according to the following standard: 1, no staining; 2, weak staining (light yellow); 3, moderate staining (yellow-brown); 4, strong staining (brown). The staining index (SI) was calculated as the product of the staining intensity score and the proportion of positive tumor cells. Using the method of assessment, we evaluated protein expression in ovarian cancer tissues by determining the SI, with scores of 0, 2, 3, 4, 6, 8, 9, 12, and 16. Samples with a SI ≥ 8 were considered as high expression and samples with a SI < 8 were considered as low expression.

### Apoptosis assay

The indicated cells were treated with CDDP (12.3 μM) for 24 h, then stained by using Annexin V/FITC Cell Apoptosis Kit (KeyGEN BioTECH, Nanjing, China) according to the manufacturer’s instructions. Briefly, indicated cells were washed with PBS and binding solution, subsequently added 5 μl of Annexin V antibody in binding buffer and addition of 2 μl PI, followed by incubation for 15 min. And flow cytometric analysis was performed with a CytoFLEX flow cytometer (BECHMAN COLTER, CA, USA) to determine the percentage of apoptotic cells. Data were analyzed using FlowJo10 (Tree Star, Ashland, Oregon, USA). The experiments were repeated at least three times.

### Western blotting (WB) analysis

According to a standard protocol [52], western blot was carried out using the following antibodies: anti-DDUP (Sino Biological, Wuhan, China), anti-GAPDH (#60004-1-Ig, Proteintech,Wuhan, China), respectively. Full and uncropped western blots were provided in Supplementary Fig. 4.

### Immunofluorescence (IF) staining

The indicated cells were plated on chamber slide cultures (Thermo Fisher Scientific, CA, USA), and then treated with anti-DDUP antibody (Sino Biological, Wuhan, China), anti-γH2AX antibody (#9718, Cell Signaling Technology, MA, USA), anti-PCNA (#2586, Cell Signaling Technology, MA, USA), anti-RAD18 antibody (#9040, Cell Signaling Technology, MA, USA), or anti-RAD51C antibody (PA5-77078, Invitrogen, USA). The photographs were taken with the Axion Vision Rel.4.6 computerized image analysis system (Carl Zeiss, Jena, Germany).

### Plasmid and transfection

The PCR-amplified human CTBP1-DT sequence was subcloned and cloned into pSin-EF2-vector to produce the complete length of CTBP1-DT. By cloning the entire length of CTBP1-DT, the DDUP ORF with FLAG-tag expression plasmid was created. CTBP1-DT/ATG1m, CTBP1-DT/ATG2m, and CTBP1-DT/ATG1/2m mutation constructs in which the putative ORF start codon in the CTBP1-DT-psin EF2 vector was mutated to ATT. The DDUP/T174D mutant were created using the QuikChange Site-Directed Mutagenesis Kit (Agilent Technologies, CA, USA). Following the manufacturer’s instructions, vectors were transfected using Lipofectamine 3000 (Thermo Fisher Scientific, CA, USA).

### MTT assay

The indicated cells were seeded in 96-well plates. Cells were exposed to the indicated concentration of CDDP 24 h and further cultured for 96 h in the fresh medium and then incubated with MTT solution (0.5 mg/ml, Sigma-Aldrich, St Louis, MO, USA) for 4 h at 37 °C. The culture medium was removed, and the cells were treated with 160 μl of dimethyl sulphoxide (Sigma-Aldrich, St Louis, MO, USA). Following the manufacturer’s instructions, measurements of absorbance were made at 490 nm using a Sunrise Microplate Reader (Tecan Sunrise, Switzerland). The CDDP half-maximal inhibitory concentration (IC50) values were determined using GraphPad Prism (Version 8.0.1). Results are representative of three independent experiments. Error bars indicate standard deviation (SD) of three biological replicates.

### Drug combination analysis

To evaluate the effects of CDDP and Berzosertib combination treatment, the indicated cells were incubated with CDDP and Berzosertib and cell viability was analyzed using MTT assays. Briefly, 3500 cells/well were seeded into 96-well plates, grown for 24 h, and treated with CDDP (0.625, 1.25, 2.5, 5, 10, 20, 40, 80 μΜ) and Berzosertib (10, 20, 40, 80, 160, 320, 640, 1280 nM) alone at serial dilutions, or combination of CDDP (12.3 μΜ) with Berzosertib (10, 20, 40, 80, 160, 320, 640, 1280 nM) for 96 h. After that, 100 ml sterile MTT dye (0.5 mg/ml, Sigma) was added to each well for 4 h at 37 °C. Then, the media were removed and 150 μl dimethyl sulfoxide (Sigma-Aldrich, St Louis, MO, USA) was added. Finally, the absorbance of each well was measured at 570 nm using an EPICS XL flow cytometer (Beckman-Colter).To evaluate the synergistic effects of CDDP and Berzosertib combinations, synergistic drug interactions were calculate with the Chou and Talalay method using Compusyn software according to non-constant ratio design between drug combinations [31, 32]. The combination index (CI) [31] for drug combination is derived according to the equation below where *n* = number of drugs, fa = fraction affected, fu = fraction unaffected.

$${\rm{CI}}=\mathop{\sum }\limits_{j=1}^{n}\frac{{({f}_{a})}_{j}}{{({f}_{u})}_{j}}$$

The resulting combination index (CI) theorem offers quantitative definition for additive effect (CI = 1), synergism (CI < 1), and antagonism (CI > 1) in drug combination.

For mechanistic studies PDOVCs#3 and PDOVCs#4 cells were treated as described above but using the most synergistic drug combination of 12.3 μΜ CDDP and 80 nM Berzosertib.

### Neutral comet assay

The comet assay was carried out in accordance with the manufacturer’s instructions (Trevigen, MD, USA) as described [53]. Briefly, after centrifugation, the indicated cells were collected and resuspended at 1 × 10^5^ cells /ml in pre-cold PBS (Ca^++^ and Mg^++^ free). Combine cells in a 1:10 (v/v) ratio with molten LMAgaros and immediately transfer 50 μl evenly onto CometSlide^TM^. Immerse slides in 4 °C lysis solution overnight after the agarose has solidified at 4 °C for added sensitivity. Following that, slides were gently immersed in 50 ml of 1 × Neutral Electrophoresis buffer for 30 min after being removed from the lysis buffer. For the gel electrophoresis, add ~850 ml 4 °C 1 × Neutral Electrophoresis buffer to the slides, and set voltage at 1 volt per cm. After then, cells were fixed with 70% (v/v) ethanol and stained with SYBRTM Gold (#S11494, Invitrogen, CA, USA). Using the plugin OpenComet v1.3.1, DNA damage was quantified for 100 cells for each experimental condition by determining tail moment. The tail moment is calculated as percent DNA in the tail multiplied by the tail length. The tail moment was normalized to the control group to obtain relative tail moment. Results are representative of three independent experiments. Statistical analysis was done using the Student’s *t* test.

### Determination of cisplatin-induced interstrand crosslinking

The determination of interstrand cross-linking was examined using a modification of single cell gel electrophoresis (comet assay). Briefly, the indicated cells were treated with CDDP (12.3 μΜ) alone or combination of CDDP (12.3 μΜ) and Berzosertib (80 nM) for 1 h. After that, cells were incubated in fresh medium for 7 h before cross-linking analysis. All CDDP-treated cells and one control were irradiated with IR (12.3 Gy) immediately before analysis to generate a random DNA strand breaks, one unirradiated control was also included. In accordance with the directions in the Comet Assay kit (Trevigen, 42150-050-K), we carried out an alkaline denaturing comet assay. For each slide, 100 cells were analyzed. Olive tail moment was obtained by using the OpenComet v1.3.1 plug in Image J [54]. The tail moment is calculated as product of percentage of DNA in the comet tail and distance between the head and tail. The presence of cross-linkings slows the migration of irradiated DNA during electrophoresis, resulting in a lower tail moment compared to control cells. The number of cross-linkings was calculated through the comparison of the tail moment of the irradiated CDDP-treated cells to the tail moment of the irradiated untreated cells and the unirradiated untreated controls. Cross-linking was calculated using the formula: % decrease in Olive Tail Moment = [1-(TMdi-TMcu)/ (TMci-TMcu)] × 100, where TMdi is the mean tail moment of drug-treated irradiated sample, TMcu is the mean tail moment of untreated, unirradiated control sample, and TMci is the mean tail moment of untreated, irradiated sample. In combination studies of CDDP and Berzosertib, the following formula was used: % decrease in Olive Tail Moment = [1-(TMdi-TMcu)/ (TMci-TMcu) + (TMdu-TMcu)] × 100, where TMdu is the tail moment of drug-treated, unirradiated samples to take into account any extra strand breaks produced by Berzosertib. Results are representative of three independent experiments.

### RNA extraction, reverse transcription, and Polymerase Chain Reaction (PCR)

Total RNA was extracted from the indicated cells using Trizol (Thermo Fisher Scientific, CA, USA) reagent, and total mRNA reverse transcription was performed according to the manufacturer’s instructions using a GoScriptTM Reverse Transcription Mix kit (Promega, Beijing, China). PCR was then conducted on the reverse-transcribed cDNA. Using the FastStart Universal SYBR Green Master (ROX; Roche, Toronto, CA), real-time q-PCR was performed and quantified in the Bio-Rad CFX qRT-PCR detection system (Applied Biosystems Inc, CA, USA). Expression data were normalized to the geometric mean of housekeeping gene GAPDH to control the variability in expression levels and calculated as 2^-[(Ct of gene) – (Ct of GAPDH)]^, where Ct represents the threshold cycle for each transcript. Primers as follows: CTBP1-DT Forward Primer: 5’-CCATCCTCTGCAGCAAGTCA -3’; CTBP1-DT Reverse Primer: 5’-CTCCGTTCTCAGTTGCCTGT-3’. Results are representative of three independent experiments.

### Targeted gene disruption by CRISPR-Cas9

The CRISPR/Cas9 system was used to generate DDUP heterozygous knockout PDOVC cells. The corresponding gRNA1 (GGTTGGTGGAGTGCACAGGCAGG) and gRNA2 (TGCACAGGCAGGGACCTCACTGG) were designed and cloned into the GV392 plasmid, respectively, by GeneChem (Guangzhou, China). In brief, lenti-CRISPR virus was introduced into 3 × 10^5^ indicated PDOVC cells. After 24 h, the infected cells were selected for 7 days with puromycin at 0.5 g/ml. Following that, #3 and #4 PDOVC/Cas9 cells were re-infected with the GV392-GFP-CTBP1-DT gRNA lentivirus at a MOI of 4 to ensure that >95% of cells were positive. Two days later, the infected cells were sorted using flow cytometry and single-cell cloned, the PDOVC#3/DDUP^-/-^-1, PDOVC#3/DDUP^-/-^-2, PDOVC#4/DDUP^-/-^-1, and PDOVC#4/DDUP^-/-^-2. PDOVC#3/DDUP^-/-^-1 and DDUP^-/-^-2 referred to PDOVC#3 single-cell clones 1 and 2, respectively. PDOVC#4/DDUP^-/-^-1 and DDUP^-/-^-2 referred to PDOVC#4 single-cell clones 1 and 2, respectively. DDUP depletion was validated by western blot.

### Xenografted tumor models

All of the animal procedures and ethical approval were approved by the Sun Yat-sen University Animal Care Committee (SYSU-IACUC-2021-000674). Before tumor cell/tissues transplantation, mice were randomized into different groups (six in each group) according to their body weight to ensure that there were weight-induced differences. To authentically mimic the ovarian cancer growth in patients, we generated a patient-derived xenografts (PDX) tumor model beneath the skin of female NOD-SCID IL-2rγ^−/−^ (NSG) mice (4–8 weeks old). In brief, subcutaneously implanted fragments (1–3 mm^3^) of freshly isolated clinical ovarian cancer patient tissues. Two weeks after the tumor transplantation, the mice received systemic administration of various agents. Every other day, the body weight and tumor volume of the mice were assessed. The tumors’ dimensions were measured with a vernier caliper. Tumor volume was calculated using the formula: *V* = 0.5 × length × width^2^. Recipient mice bearing ~0.2 cm^3^ size of tumor were intraperitoneally treated with vehicle (control), Carboplatin (50 mg/kg), Berzosertib (60 mg/kg), or Carboplatin (50 mg/kg) combined with Berzosertib (60 mg/kg), three times per week up to 6 weeks. In the subcutaneously tumor model, cells stably expressing DDUP/WT or DDUP/mutant were subcutaneously inoculated into female NOD-SCID mice. When the tumor became palpable, the mice treated with combination of vehicle and Carboplatin (50 mg/kg), or combination of Carboplatin (50 mg/kg) and Berzosertib (60 mg/kg), three times per week up to 6 weeks.

These mice were immediately put to death at the conclusion of the treatment, and the tumors were collected, weighed, measured, and ready for additional examination. Tumor sections were stained by IHC using anti-DDUP antibody (Sino Biological, Wuhan, China), anti-ATR pS428 antibody (PA5-39773, Thermo Fisher Scientific, CA, USA), anti-γH2AX antibody (#9718, Cell Signaling Technology, MA, USA), anti-Ki-67 antibody (PA5-19462, Thermo Fisher Scientific, CA, USA) or TUNEL analysis (In Situ Cell Death Detection Kit, TMRred, Roche Applied Science) according to the manufacturer’s protocol. The images were captured using the AxioVision Rel.4.6 computerized image system (Carl Zeiss, Jena, Germany).

### Fluorescence recovery after photo-bleaching (FRAP)

Fluorescence recovery after photo-bleaching (FRAP) was carried out using a Carl Zeiss LSM 880 with Airyscan confocal microscope (Carl Zeiss, Jena, Germany) and a 63× oil (NA1.4) objective. The indicated cells transfected with GFP-RAD18 alone or co-transfected with DDUP plasmids were cultured on 15 mm glass-bottom dishes (NEST, Wuxi, China). Following the acquisition of two prebleach images, the GFP-RAD18 fluorescence was then photo-bleached using scans with a 488 nm argon laser at 100% power. Images were captured at 400 s intervals for post-bleached recovery recording, and the fluorescence intensity within a specific region was measured every 80 s at 20% laser power. After subtracting the background, the fluorescence intensity was normalized to the pre-bleached signal. Results are representative of three independent experiments. Data were plotted using GraphPad Prism 8 software.

### Statistical analysis

GraphPad Prism v.8.0.1 for Windows and Microsoft Excel 2016 were used for the statistical analysis. The average and standard deviation of at least three biological replicates are used to represent experimental data. The combination index was calculated using the method of non-constant ratio drug combination proposed by Chou and Talalay [31]. Statistical significance was defined as a *P*-value of 0.05 or less. Unpaired, two-tailed Student’s *t* tests were used for parametric data. The two-sided Mann-Whitney test was used for non-parametric data. A chi-squared test was used to analyze the relationship between DDUP expression and the clinic pathological characteristics. Statistical analyses were performed using the SPSS 11.0 statistical software package.

## Supplementary information

Supplementary Figure 1

Supplementary Figure 2

Supplementary Figure 3

Original Data File

Supplementary data

Checklist

## Data Availability

The data supporting the findings from this study are available within the article file and its supplementary information. Uncropped images of Immunoblotting were provided as supplementary material.
