# Supplementary figures and images for: The DDUP protein encoded by the DNA damage-induced CTBP1-DT lncRNA confers cisplatin resistance in ovarian cancer
Source: Cell Death Dis. 2023 Aug 26;14(8):568. doi: 10.1038/s41419-023-06084-5 (PMC10460428; doi:10.1038/s41419-023-06084-5)

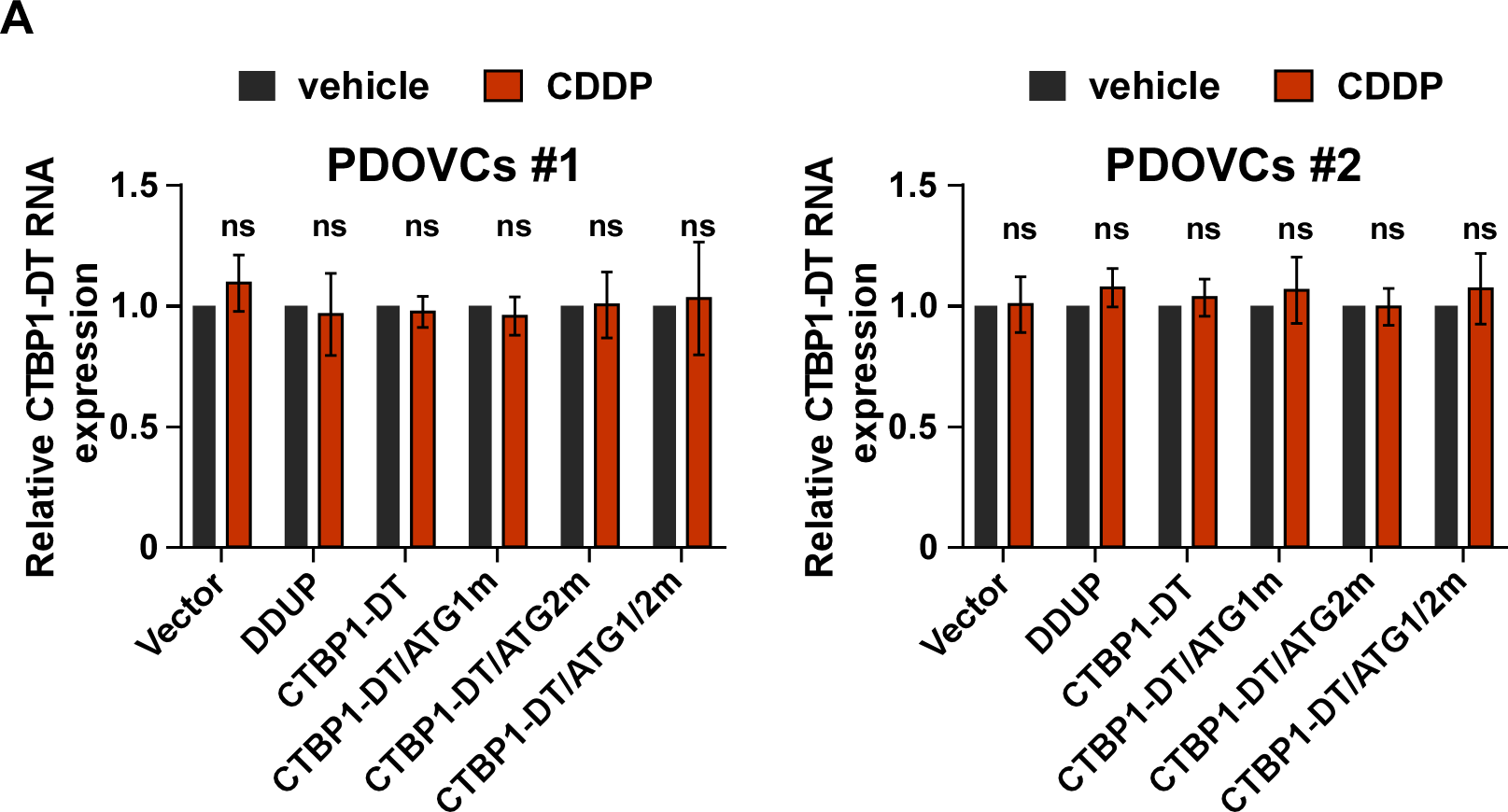

Supplement: Supplementary file 1 — Supplementary Figure 1 [file 41419_2023_6084_MOESM1_ESM.tif]

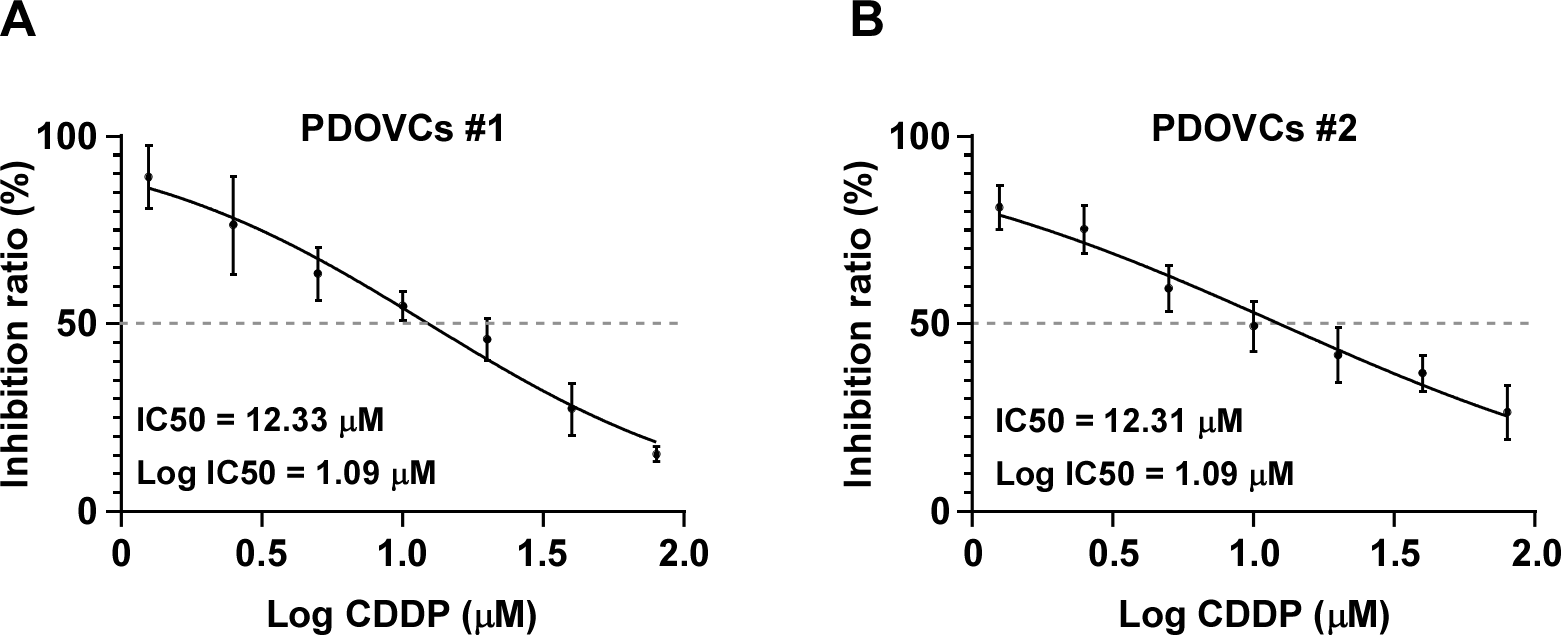

Supplement: Supplementary file 2 — Supplementary Figure 2 [file 41419_2023_6084_MOESM2_ESM.tif]

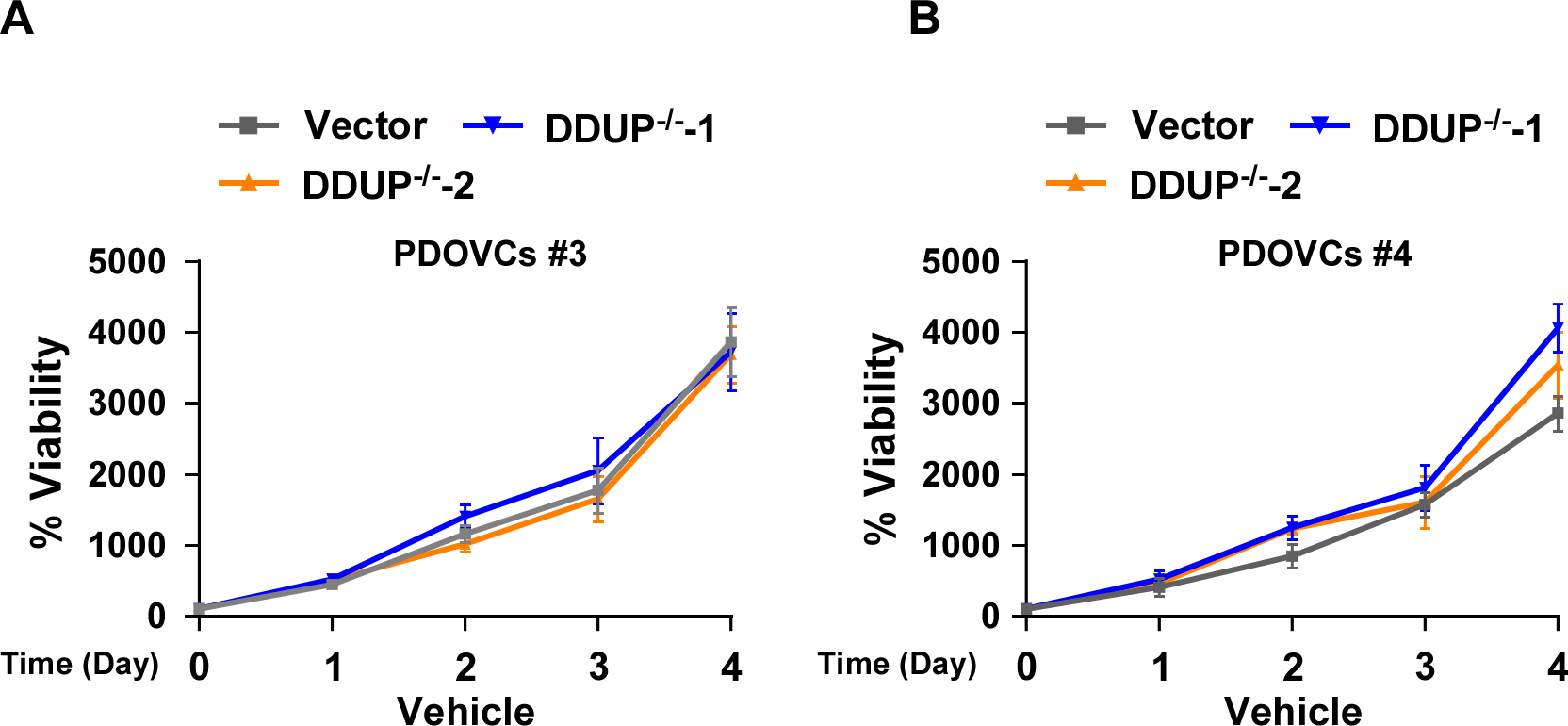

Supplement: Supplementary file 3 — Supplementary Figure 3 [file 41419_2023_6084_MOESM3_ESM.tif]

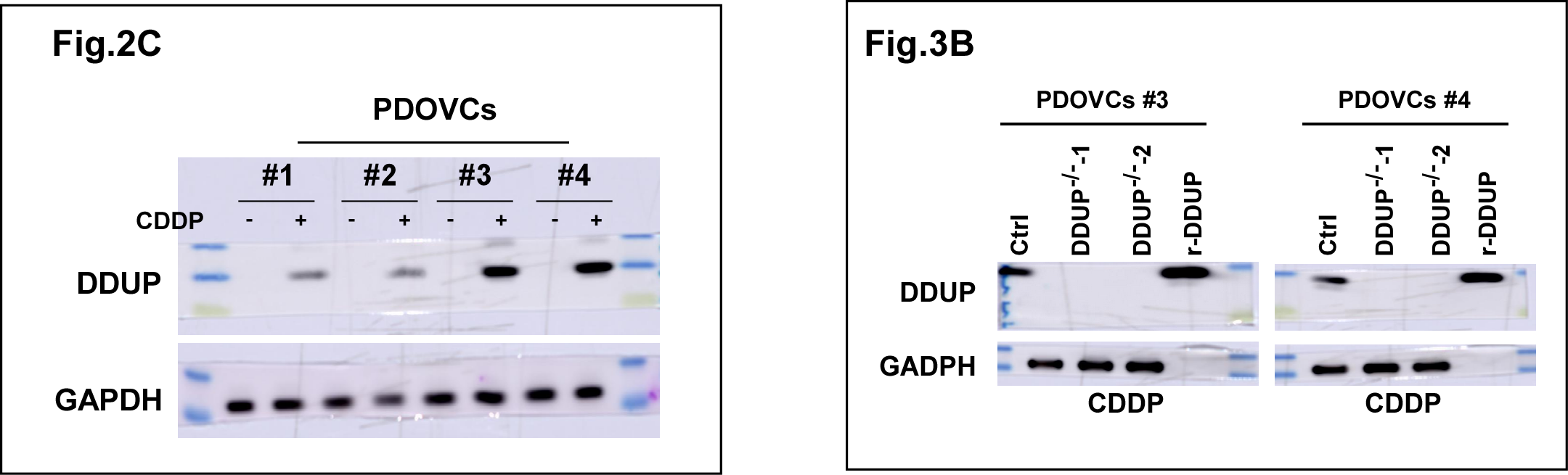

Supplement: Supplementary file 4 — Original Data File [file 41419_2023_6084_MOESM4_ESM.tif]
