# Supplementary material for: The DDUP protein encoded by the DNA damage-induced CTBP1-DT lncRNA confers cisplatin resistance in ovarian cancer
Source: Cell Death Dis. 2023 Aug 26;14(8):568. doi: 10.1038/s41419-023-06084-5 (PMC10460428; doi:10.1038/s41419-023-06084-5)
Supplement: Supplementary file 5 — Supplementary data [file 41419_2023_6084_MOESM5_ESM.docx]

**Supplementary information for**

**The DDUP protein encoded by the DNA damage-induced CTBP1-DT lncRNA confers cisplatin resistance in ovarian cancer**

Liangliang Ren ^1,^ **^#^**, Xingrong Qing ^2,^ **^#^**, Jihong Wei ^2,^ **^#^**, Haixin Mo ^1^, Yuanji Liu ^5^, Yaofeng Zhi ^1^, Wenjie Lu ^1^, Mingzhu Zheng ^1^, Weijian Zhang ^2^, Yuan Chen ^2^, Yuejiao Zhang ^2^, Taijin Pan ^2^, Qian Zhong ^2^, Ronggang Li ^3^, Xin Zhang ^1^, Xiaohong Ruan ^1, 2,^ *, Ruyuan Yu ^4,^ *, Jun Li ^1, 5,^ *

*** Correspondence to:** Jun Li, E-mail: [lijun37@mail.sysu.edu.cn](mailto:lijun37@mail.sysu.edu.cn)

Ruyuan Yu, E-mail:[yury6@mail2.sysu.edu.cn](mailto:yury6@mail2.sysu.edu.cn)

Xiaohong Ruan, E-mail: [ruanxiaohong@jmszxyy.com.cn](mailto:ruanxiaohong@jmszxyy.com.cn)

**Supplementary Fig. 1. CDDP treatment has no effect on the expression of CTBP1-DT lncRNA. A.** Real-time analysis of the expression of CTBP1-DT lncRNA in PDOVCs#1 and PDOVCs#2 treated with the vehicle or CDDP. GAPDH served as the loading control. The error bars represent the mean ± SD of data from three independent experiments. *P < 0.05, **P < 0.01, ***P < 0.001.

**Supplementary Fig. 2. The half-maximal inhibitory concentration (IC50) growth inhibition values of CDDP in PDOVCs#1 and PDOVCs#2. A–B.** PDOVCs#1 **(A)** and PDOVCs#2 **(B)** were treated with various concentration of CDDP and subjected to MTT assays. X-axis is a log10 scale. IC50 is shown as a dotted line in the middle of the graph. Inhibition ratio (%) = (OD control- OD sample) / (OD control) × 100%. The error bars represent the mean ± SD of data from three independent experiments. *P < 0.05, **P < 0.01, ***P < 0.001.

**Supplementary Fig. 3. DDUP had no effect on cell survival in the absence of induction of DNA damage. A–B.** Survival rates of **(A)** PDOVCs#3 **(B)** PDOVCs#4 control cells and DDUP^-/-^ PDOVCs#3 and PDOVCs#4 were determined by MTT assays following treatment with the vehicle at the indicated time intervals. The error bars represent the mean ± SD of data from three independent experiments. *P < 0.05, **P < 0.01, ***P < 0.001.

**Supplementary Fig. 4. Uncropped images of immunoblotting.**
